# Acidochromogenicity is a common characteristic in nontuberculous mycobacteria

**DOI:** 10.1186/1756-0500-4-466

**Published:** 2011-10-29

**Authors:** Beatrice Saviola, Jeffrey Felton

**Affiliations:** 1 Basic Medical Sciences, College of Osteopathic Medicine, Western University of Health Sciences, 309 E. Second St., Pomona, CA 91766, USA

## Abstract

**Background:**

An acidic environment is something likely encountered by mycobacteria in the environment or in a human host. Previously mycobacterial species had been known to produce carotenoid pigments in response to light or constitutively.

**Results:**

We have tested the ability of various mycobacteria to grow on solid agar plates of differing acidity, and have shown that many species of mycobacteria previously thought to not produce pigment are pigmented when exposed to acidic stress. The *Mycobacterium smegmatis *promoter region upstream of the genes homologous to those of other mycobacterial species known to code for proteins involved in carotenoid biosynthesis was found to be upregulated under acidic stress.

**Conclusions:**

Mycobacterial species can produce pigment in response to conditions not previously known to induce chromogenicity in mycobacteria. In addition many mycobacterial species previously thought to not produce pigment are actually chromogenic under acidic conditions.

## Background

For the past 51 years the Runyon System has divided nontuberculous mycobacteria into four groups based on the production of pigment either constitutively (scotochromagens), in response to light (photochromogens), not at all (nonchromogens), or growth rate [1-7]. Using this knowledge, medical microbiologists have differentiated among many mycobacterial species. We have identified a number of mycobacterial species previously thought to be nonchromogens which produce pigment in response to acidic stress, a condition likely encountered in the environment and within the human host and not previously known to induce chromogenicity.

Environmental stresses have been known for some time to induce production of microbial pigments. These stresses include hyper osmolarity, UV light exposure, methanol exposure, elevated temperature, and low iron [8-10]. Many of these stresses result in increased oxidative damage within the microbial cell and pigments have been shown to decrease the overall level of reactive oxygen intermediates [11,12]. Examples of pigments produced include melanin and carotenoid compounds of which carotenoids contain conjugated double bonds which are efficient at scavenging reactive oxygen intermediates and are known to be incorporated into the microbial cell membrane altering fluidity and potentially serving as a barrier to environmental stress. Some pathogens produce carotenoids constitutively such as *Staphylococcus aureus*. This pigment has been known to promote virulence through its antioxidant activity and helps the pathogen to withstand neutrophil killing [13]. Compounds that impair synthesis of the pigment also interfere with *S. aureus *virulence [14]. Pathogens that produce pigment inducibly or constitutively may withstand both environmental stress as well as stresses *in vivo*. Many bacteria which cause disease in humans and produce pigment such as *Vibrio cholerae *as well as *Mycobacterium avium *are also present in aquatic environments where they must withstand environmental extremes [8]. Pigment production may be a vital microbial defense that increases environmental persistence and *in vivo *pathogenesis.

## Results

**Figure 1:**
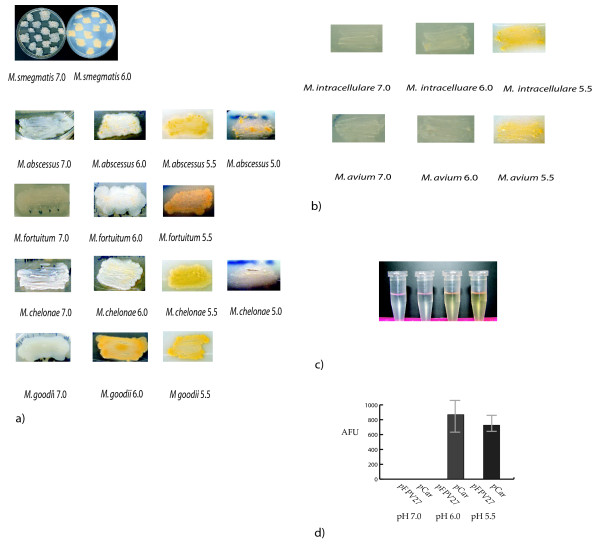
**a) *M. smegmatis*, *M. abscessus*, *M. fortuitum subsp. fortuitum*, *M. chelonae*, and *M. goodii *were patched onto 7H10 agar media (Difco) at pH 7.0, 6.0, 5.5 or 5.0 to determine pigment production**. b) *M. avium intracellulare *and *M. avium subsps. avium *were patched onto 7H10 agar media at pH 7.0, 6.0, and 5.5 to determine pigment production. c) From left to right: acetone only, *M. smegmatis *pH 7.0 extracted w/acetone, *M. smegmatis *pH 6.0 extracted w/acetone, *M. smegmatis *pH 5.5 extracted with acetone. d) Fluorescence specific activity units of *M. smegmatis *bearing empty reporter plasmid *pFPV27 *or the *M. smegmatis *probable carotenoid promoter upstream of *gfp *(*pCar*) at pH 7.0, 6.0 or 5.5.

We have tested for acid-chromogenicity in five rapidly growing mycobacterial species of the Runyon Class IV designation previously thought to not produce pigment. *Mycobacterium abscessus*, *Mycobacterium fortuitum subsp. fortuitum*, *Mycobacterium chelonae*, *Mycobacterium smegmatis*, and *Mycobacterium goodii *were tested. Isolated colonies of mycobacteria were patched onto 7H10 agar media (Difco) adjusted to pH 5.5 or 6.0 with HCl, or pH 7.0 with NaOH and containing 10% ADC (bovine serum albumin, dextrose and NaCl) and 0.2% glycerol and were incubated for 72 hours at 37°C in the dark. Agar plates were inspected visually to determine pigment production. All bacterial species tested did not produce pigment at pH 7.0, but did produce pigment at pH 5.5 (Figure 1a). Only two of the species, *M. smegmatis *and *M. goodii*, produced the pigment at pH 6.0, and are genetically closely related (Figure 1a). Two mycobacterial species, *M. chelonae *and *M. abscessus *were tested at pH 5.0 and only *M. abscessus *continued to produce pigment at this low pH. We also tested two slow growing species of Runyon class II designation thought to be nonchromogens *Mycobacterium avium intracellulare *and *Mycobacterium avium avium*. These two species produced pigment at pH 5.5 but failed to do so at pH 7.0 and 6.0. Again acid pigment formation has not been previously described for an *M. avium *species (Figure 1b).

To investigate the nature of the pigment *M. smegmatis *was grown on pH 5.5, 6.0 or 7.0 agar plates and extracted with acetone. Acetone extracts from *M. smegmatis *grown on pH 5.5 and pH 6.0 agar plates contained a vibrant yellow color while acetone used to extract *M. smegmatis *grown on pH 7.0 plates remained colorless as did an acetone only control indicating the pigment is likely present in the cell wall (Figure 1c). We found an absorbance maximum at pH 6.0 and 5.5 of 40 nm but no appreciable absorbance at pH 7.0. β-carotene has a maximal absorbance at 450 nm.

*M. marinum *has been extensively studied as to its photochromogenicity [15,16]. Using information that was available for the well-studied *M. marinum*, we identified genes within the *M. smegmatis *genome that are likely responsible for pigment production. The genes are present within a probable operon containing six open reading frames (TIGR). These genes appear to produce enzymes that catalyze various steps in the carotenoid biosynthesis pathway. We synthesized oligonucleotide primers *car1 *(5'ATGTATGGTACCTCGGGCCGCCCGGCAAAGCACG3') and *car2 *(5'ATGTATGGATCCTGGCCGACGAGACCGCGCAGGTG3') to bind and amplify a DNA region upstream of the probable operon. This DNA includes the putative 133 bp promoter as well as an additional 100 bp 5' to the operon resulting in a 233 bp DNA fragment. This region was inserted upstream of the gene for the green fluorescent protein (*gfp*) from the jelly fish *Aequorea victoria *in the mycobacterial shuttle plasmid *pFPV27 *[17]. This resultant mycobacterial shuttle plasmid was then inserted into *M. smegmatis *and individual mycobacterial colonies were patched onto 7H10 agar plates at pH 5.5, 6.0, and 7.0. Patched bacteria were grown for 24 hours, were scraped from the agar plates, and resuspended into 7H9 broth media. These mycobacteria were then vortexed with 0.5 mm glass beads to break up bacterial clumps, and were allowed to settle for 5 min. Mycobacteria remaining suspended in the growth media were then diluted so that each sample possessed the same approximate optical density. Fluorescence was measured on a Turner Designs fluorometer. Fluorescence specific activity was determined by dividing fluorescence values by optical density values at 600 nm. The carotenoid promoter was upregulated in *M. smegmatis *when grown at pH 5.5 and pH 6.0, but remained silent when grown at pH 7.0 (Figure 1d).

## Discussion

Acidic stress is likely encountered within environmental and clinical habitats of mycobacteria. *M. smegmatis *was first identified from the genital secretions of humans but known to only infrequently cause human disease [18]. It has, however, been associated with skin and soft tissue infections, bursitis, catheter related infections, and disseminated infections in the immunocompromised [19-24]. Within the human body, macrophages invariably phagocytose *M. smegmatis *and the phagocytic compartments undergo acidification. Within phagosomes pH initially drops to pH 5.5 or lower and then rebounds to 6.5. Within activated macrophages, however, pH inside phagosomes can drop as low as 4.5 [25-27]. Thus mycobacterial species are exposed to acidic pH's intracellularly that can induce pigment formation. In addition acidity can be encountered extracellularly. Acidity normally found in genital secretions, or on the skin surface could serve as an environmental stressor for *M. smegmatis *[28]. Thus, there are ample opportunities in the environment, both natural and clinical, to stimulate pigment production.

Environmental mycobacteria such as *M. avium *sp., *M. chelonae*, *M. abscessus*, and *M. fortuitum *may encounter acidity in stagnant water, certain brooks and streams, or in the soil. It is also possible that environmental protozoa can phagocytose these environmental mycobacteria and expose them to acidic stress within their phagocytic compartments. This environmental acidic priming may make environmental mycobacteria more resistant to acidity encountered on or within the human body especially if induction of pigment production results in increased survival during acidic stress.

*M. smegmatis *and *M. goodii *are considered nonchromogens, but it has been shown that they can produce a late developing pigment that appears upon extended growth of the microorganisms (7-10 days) and this may be related to acid induction. Auto acidification is an explanation for this phenomenon that incorporates the idea that acidity induces chromogenicity in these species. This process may take place within the microenvironment of the colony as the bacteria metabolize nutrients increasing local proton concentrations resulting in late acidification that stimulates pigment production.

Pigment production was not tested in *Mycobacterium tuberculosis*. In previous studies pigment appeared upon extended growth of *M. tuberculosis *at low oxygen tension and pigment was visualized by chromatography [29]. It is intriguing to speculate that *M. tuberculosis *also induces pigment in response to acidic stress encountered within the phagosome of macrophages or the centers of caseating granulomas. Further studies need to be performed with *M. tuberculosis *growing on agar media at acidic pH's to determine if pigment is produced under these conditions.

While many mycobacterial species have previously been identified to produce pigment in response to light or constitutively, acid induced pigment production had not been previously described. Seven previously classified nonchromogens produce pigment in response to acidity. It remains to be determined if other nonchromogenic atypical mycobacteria also produce pigment in response to acidity. In addition it will be of interest to determine if mycobacterial species previously identified as photochromogens are also acidochromogens, or if scotochromogens produce an even greater quantity of pigment in response to acidity. Thus more studies need to be performed with other mycobacterial species at a variety of pHs to determine how prevalent acidochromogenicity is in mycobacterial species.

## Conclusions

The 7 mycobacterial species tested in this study produce pigment in response to acidity. Additional investigation may reveal acidic conditions where pigments are differentially produced. *M. smegmatis *and the closely related *M. goodii *produce pigment at pH 6.0, production at a higher pH than other species tested. In addition the closely related *M. chelonae *and *M. abscessus *were tested at pH 5.0. While *M. chelonae *ceases to produce the pigment, *M. abscessus *continues to produce it. Other differential patterns of pigment production may be identified for the other acidochromogenic species and may ultimately lead to a manner by which to identify atypical mycobacteria rapidly in an environment lacking sophisticated biomedical equipment such as in developing countries. Visual inspection of mycobacterial growth for pigment production could allow clinical laboratory workers an initial tentative identification before more labor intensive methods are employed to identify mycobacterial species [30]. This could provide time and resource saving information before performing further biochemical, nucleic acid probe, or chromatographic tests to positively identify mycobacterial species depending on the level of resources available.

It remains to be determined what environmental advantage pigment production confers on mycobacteria in response to acidity. Pigments may aid mycobacteria in resisting cellular damage due to acidic stress. Alternatively acidity may prime pigment production which combats a concurrent stress such as oxidative stress likely encountered *in vivo *or in the environment.

## Methods

### Strains and Media

The *Mycobacterium smegmatis *strain MC2155 (American Type Cell Culture number-ATCC 700084), *Mycobacterium chelonae *(ATCC 14472), *Mycobacterium fortuitum subsp. fortuitum *(ATCC 11440), *Mycobacterium abscessus *(ATCC 23016), *Mycobacterium goodii *(ATCC BAA-955), *Mycobacterium avium subsp. avium *(ATCC 19075), and *Mycobacterium intracellulare *(ATCC 19077) were used in the experiments. For exposure to acidic stress on agar plates, 7H10 media (Difco) was adjusted to pH 5.5 or 6.0 with HCl and then autoclaved. Ten percent ADC was added before pouring the plates.

### Acid Induction on Solid Media

Isolated colonies of mycobacteria were patched onto 7H10 agar media at pH 5.0, 5.5, 6.0, or pH 7.0 containing 10% ADC (bovine serum albumin, dextrose and NaCl) and 0.2% glycerol and were incubated for 72 hours at 37°C in the dark. Agar plates were inspected visually to determine pigment production.

### Extraction of Pigment

*M. smegmatis*, was patched and grown on 7H10 agar plates + 10% ADC and 0.2% glycerol at pH 7.0, 6.0, or 5.5 for 72 hours. Mycobacteria were scraped from the plates, resuspended in 1 ml of acetone, and vortexed 10 times during a 15 min interval. The same approximate amount of bacteria was used for each extraction. Mycobacteria were centrifuged and the supernatant was collected. Absorbance of the extracts was measures on a spectrophotometer between 375 and 550 nm at 10 nm intervals.

Acid Induction of the Promoter Driving Carotenoid Biosynthesis Genes in *M. smegmatis*

Using information that was available for the well-studied *M. marinum*, we identified genes within the *M. smegmatis *genome that are homologous to genes from *M. marinum *responsible for pigment production. The genes are present within a probable operon containing six open reading frames (TIGR) and appear to code for enzymes that catalyze various steps in the carotenoid biosynthesis pathway. We synthesized oligonucleotide primers car1 (5'ATGTATGGTACCTCGGGCCGCCCGGCAAAGCACG3') and car2 (5'ATGTATGGATCCTGGCCGACGAGACCGCGCAGGTG3') to bind and amplify a DNA region upstream of the probable operon. This DNA includes the putative 133 bp promoter as well as an additional 100 bp 5' to the operon resulting in a 233 bp DNA fragment. This region was inserted upstream of the gene for the green fluorescent protein (*gfp*) from the jelly fish *Aequorea victoria *in the mycobacterial shuttle plasmid *pFPV27 *[17]. This resultant mycobacterial shuttle plasmid was then inserted into *M. smegmatis *and individual mycobacterial colonies were patched onto 7H10 agar plates at pH 5.5, 6.0, and 7.0. Patched bacteria were grown for 24 hours, were scraped from the agar plates, and resuspended into 7H9 broth media. These mycobacteria were then vortexed with 0.5 mm glass beads to break up bacterial clumps, and were allowed to settle for 5 min. Mycobacteria remaining suspended in the growth media were then diluted so that each sample possessed the same approximate optical density. Fluorescence was measured on a Turner Designs fluorometer. Fluorescence specific activity was determined by dividing fluorescence values by optical density values at 600 nm.

## Competing interests

The authors declare that they have no competing interests.

## Authors' contributions

BS carried out all experiments delineated in the manuscript. JF contributed by comments on experimental design and manuscript preparation. All authors have read and approved the final manuscript.
